# Glycosylation in Axonal Guidance

**DOI:** 10.3390/ijms22105143

**Published:** 2021-05-13

**Authors:** Sampada P. Mutalik, Stephanie L. Gupton

**Affiliations:** Department of Cell Biology and Physiology, University of North Carolina, Chapel Hill, NC 27514, USA; mutaliksampada@med.unc.edu

**Keywords:** axonal guidance, glycosylation, glycosaminoglycan, hyaluronan, heparan sulfate proteoglycan, chondroitin sulfate, repulsion, attraction, chemotaxis, haptotaxis

## Abstract

How millions of axons navigate accurately toward synaptic targets during development is a long-standing question. Over decades, multiple studies have enriched our understanding of axonal pathfinding with discoveries of guidance molecules and morphogens, their receptors, and downstream signalling mechanisms. Interestingly, classification of attractive and repulsive cues can be fluid, as single guidance cues can act as both. Similarly, guidance cues can be secreted, chemotactic cues or anchored, adhesive cues. How a limited set of guidance cues generate the diversity of axonal guidance responses is not completely understood. Differential expression and surface localization of receptors, as well as crosstalk and spatiotemporal patterning of guidance cues, are extensively studied mechanisms that diversify axon guidance pathways. Posttranslational modification is a common, yet understudied mechanism of diversifying protein functions. Many proteins in axonal guidance pathways are glycoproteins and how glycosylation modulates their function to regulate axonal motility and guidance is an emerging field. In this review, we discuss major classes of glycosylation and their functions in axonal pathfinding. The glycosylation of guidance cues and guidance receptors and their functional implications in axonal outgrowth and pathfinding are discussed. New insights into current challenges and future perspectives of glycosylation pathways in neuronal development are discussed.

## 1. Introduction

The dynamic, motile tips of axons, called growth cones, lead growing axons in the developing nervous system. How millions of axons navigate through the complex environment of the nervous system to form specific, synaptic connections has intrigued generations of neurobiologists. The scientific journey began over 100 years ago when Ramon and Cajal proposed the chemotrophic theory, suggesting that motile axonal tips respond to chemotropic cues in the environment [1]. The chemotrophic theory proposed by Cajal was confirmed in numerous studies with the discovery of classic guidance molecules [2]. Over the years, a roadmap for growing axons to form a well-regulated synaptic network has been charted, through the identification of many guidance molecules and extracellular matrix proteins. Stereotypy of the neuronal connections is dictated by the expression patterns and spatial distributions of the guidance cues and their receptors, which form a blueprint for neuronal connections. Series of in vitro and in vivo studies have revealed molecular mechanisms regulating the interaction of guidance molecules with their cognate receptors and the downstream cellular signalling that together coordinate growth cone motility [3]. Classically, axonal guidance cues belong to four major classes: netrins, semaphorins, slits, and ephrins [4,5]. These guidance cues induce a diversity of growth cone responses through different types of receptor engagement. Beyond the classical guidance cues, many additional factors like NGF (Nerve growth factor), BDNF (brain-derived neurotrophic factor), NT3 (neurotrophin-3), and morphogens including sonic hedgehog (Shh), Wnt, and bone morphogenic protein 7 (BMP7) have been implicated in axonal guidance [4]. 

**Figure 1:**
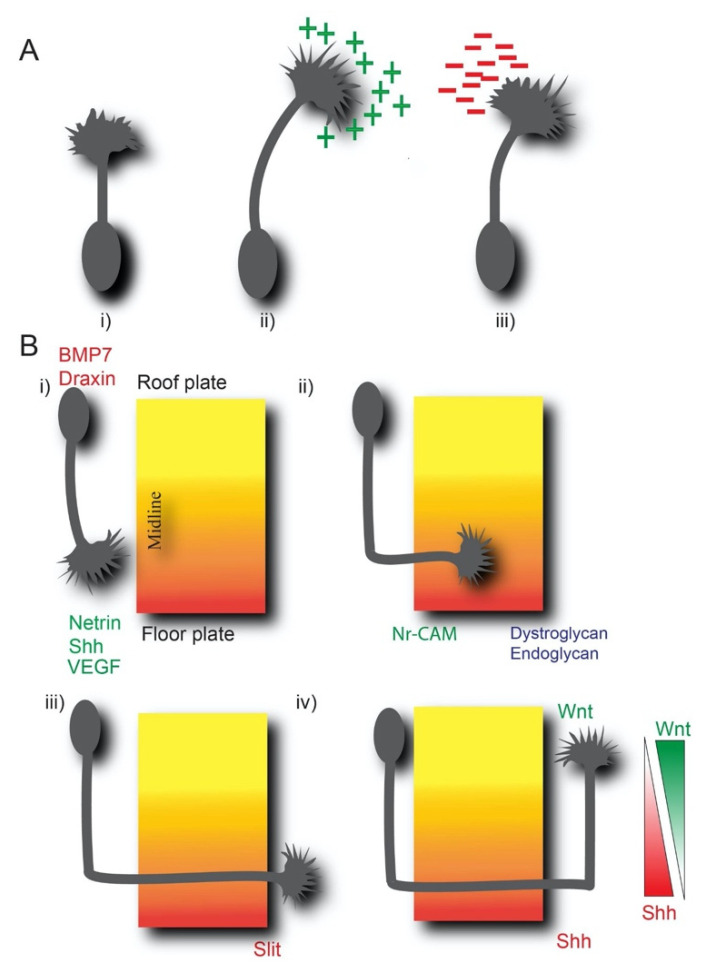
Overview of axonal guidance. (**A**) Growth cone (i) extends and turns toward the attractive cues like netrin, shh (ii) and repelled by repulsive cues like slits, semaphorins (iii). Attractive and repulsive cues are shown with + (green) and –(red), respectively. (**B**) Short-range and long-range guidance within the spinal cord. (i) Axons are repelled by BMP7 and draxin from the roof plate. Shh and netrin-1 attract axons toward the floor plate by long-range attraction. Netrin-1 produced by neural progenitor cells has also been proposed to guide commissural axons locally (not shown here) while whether floor plate netrin is dispensable or not is debated [9]. (ii) Axons enter the floor plate through short-range guidance, for example, through Nr-CAM. (iii) Slit allows axons to exit the floor plate. (iv) Axons move rostrally through the repulsive gradient of Shh (acts as an attractive cue pre-crossing and repulsive cue post-crossing) and attractive gradient of Wnt. Examples of glycosylated proteins regulating adhesion at the midline are shown (dystroglycan, and endoglycan). Receptors and multiple other guidance cues e.g., semaphorins involved in regulating midline crossing that are beyond the scope of this review are not shown here.

Growth cones extend towards attractive cues (Figure 1A i, ii) and move away from, or collapse, in response to repulsive cues (Figure 1A iii) via ligand and engagement with specific receptors. For example, the netrin receptor DCC (deleted in colorectal cancer) is involved in netrin-mediated attraction, whereas the receptor UNC-5 heterodimerizes with DCC to induce repulsion to netrin [6]. Further, synergy or crosstalk between guidance cues increases the complexity of axon guidance pathways. Some guidance molecules are secreted proteins that form long-range diffusive gradients to promote chemotaxis, whereas other cues are attached to extracellular surfaces and presented as adhesive or localized cues that promote short-range haptotaxis. Dual functions have been proposed for some guidance cues such as netrin, but the mechanistic details of such a dichotomy are not well defined [6,7]. Midline crossing in the developing spinal cord is an excellent example wherein long-range and short-range guidance mechanisms operate coordinatively (Figure 1B). dl1 commissural axons are attracted by long-range diffusive attractants like netrins and Shh towards the floor plate and are repelled by BMP7 and draxin from the roof plate (Figure 1B i) [8]. At the midline, commissural axons enter the floor plate (Figure 1B ii) through short-range attraction to Nr-CAM (Neural-glia related cell adhesion molecule). After crossing the midline, short-range chemo-repulsion from slits causes axons to exit the floor plate (Figure 1B iii). Then axons extend rostrally in response to opposing gradients of attractive Wnt and repulsive Shh, respectively (Figure 1B iv). Responsiveness to the guidance cues is regulated by the surface expression of receptors (not discussed here). There are multiple additional cues like semaphorins and vascular endothelial growth factor (VEGF) involved in midline crossing, as discussed in [7]. Largely, aforementioned guidance cues are conserved in the developing brain and spinal cord with similar guidance mechanisms.

Intriguingly, defects in axonal guidance found in different vertebrate and invertebrate systems originate from perturbations of a handful of guidance molecules [10]. Only a few novel candidate cues have been identified in recent years. For example, draxin was identified as a novel chemo-repulsive cue with no homology to classical guidance cues [7]. The postsynaptic transmembrane protein lasso was found to attract mouse neuronal growth cones via the G-protein coupled receptor (GPCR) latrophilin-1 [11]. Another recently identified guidance cue is the lipid-based guidance molecule lyso-phosphatidyl-*b*-D-glucoside (LysoPtdGlc). Although originally not considered a guidance cue due to its ubiquitous presence, recently, LysoPtdGlc released by radial glial cells was shown to repel sensory axons in the chick spinal cord [12].

Despite the identification of novel guidance cues and mechanisms, how a limited number of cues generate the complex diversity of axonal trajectories remains a fascinating puzzle. The current understanding of the cellular and developmental regulation of axonal guidance pathways has been discussed extensively in recent reviews [4,7,13,14,15]. Beyond these mechanisms, posttranslational modifications contribute significantly to regulating axon guidance pathways [7]. Glycosylation offers an additional means to diversify axon guidance pathways. In this review, we focus on this specific posttranslational modification and its impact on axonal guidance.

Post-translational modifications (PTMs) of proteins are frequently occurring mechanisms that the regulate biological functions of proteins [16]. Amongst the different types of PTMs, glycosylation is a ubiquitous, though relatively understudied protein modification, and important for protein folding, stability, and trafficking [17,18]. Glycosylation is the process of covalently attaching sugar moieties onto proteins and lipids. The process of glycan transfer occurs in the Golgi and endoplasmic reticulum and is mediated by glycosyltransferases and reversed by glycosidases, which remove them to form different glycoconjugates. The type of glycan structures formed depends on substrate availability and enzymes present. The molecular processes of glycan synthesis were recently reviewed [18].

More than 50% of mammalian proteins are predicted to undergo glycosylation [19]. The mammalian genome encodes evolutionarily conserved glycosylating enzymes and their substrates [20]. Defects in glycosylation lead to several disorders collectively known as congenital disorders of glycosylation (CDG) [20,21]. Recently, mass spectroscopy (MS)-based analysis of the mouse brain demonstrated that multiple proteins, including axonal guidance cues and receptors, undergo glycosylation during neuronal development [19]. However, the functional implications of these modifications are not completely understood [22]. Glycosylation of proteins in neuronal development and diseases has been discussed extensively in recent review articles including the role of proteoglycans in axon guidance [23], glycan structures in neuronal development [24], and the functions of the sugar code in neuronal physiology [17]. This review specifically focuses on the current understanding and future perspectives of glycosylation in axon guidance and migration.

Three major classes of glycosylation are discussed: First, N-linked glycosylation, in which glycans are linked to proteins through the nitrogen of asparagine at Asn-X-Ser/Thr motifs (where X denotes any amino acid except proline). Second, O-linked glycosylation, in which glycans are linked through the oxygen of serine or threonine at no specific sequon. Third, glycosaminoglycans (GAGs), wherein unbranched polysaccharides made of repeating disaccharide units are attached to proteins through O-linked glycosylation to form a proteoglycan. A typical N-glycan or O-glycan generally has 5–12 monosaccharides, whereas GAGs can have more than 80 sugars [18]. N-glycans are further classified into high mannose N-glycan, hybrid N-glycan, and complex N-glycan that are less processed; high mannose containing glycan, partially processed N-glycan, and processed N-glycan, respectively. Apart from these major classes, C-linked glycosylation (glycan linked to the C2 carbon of tryptophan), GPI anchors (short glycolipids that link proteins to the cell membrane), and glycosphingolipids (sphingolipids with attached glycan moieties) have also been reported in development and diseases [18]. The classification of glycoproteins is not mutually exclusive, multiple proteins are both N and O-glycosylated. In the following sections, we discuss the N and O-glycans, glycosaminoglycans and their functions in axonal guidance and neuronal migration.

## 2. N-Linked and O-Linked Glycosylation in Axonal Guidance

N-glycosylation of proteins regulates cell adhesion, trafficking, signalling, and endocytosis in different cell types [25]. N-glycosylating enzymes are ubiquitously expressed in the developing and adult nervous system and have multiple substrates. Glycoproteomic analysis of the mouse brain identified multiple proteins to be glycosylated including ion channels proteins, cell adhesion proteins, synaptic proteins, and axonal guidance molecules [19]. Inactivation of glycosyltransferases genes that link sugar moieties to proteins and lipids have highlighted functions of N- glycosylation in the nervous system [22]. For example, the conversion of high mannose to hybrid and complex N-glycan is mediated by glycosyltransferses encoded by *Mgat1* and *Mgat2* genes, respectively. Brain-specific inactivation of *Mgat1* resulted in a deficiency of certain neuronal glycoproteins and consequently, apoptosis in neurons, highlighting the functions of hybrid N-glycans. On the other hand, complex N-glycans appeared to be less critical for survival [26]. A more recent glycomic study however demonstrated the spatial and temporal diversity of both hybrid and complex glycan structures in human and mouse brain samples, highlighting diverse potential functions of glycosylation in neuronal development [27]. With a multitude of glycan structures identified in this study, future identification of their functions will help fill the knowledge gaps in the field.

Similar to N-glycosylation, O-linked glycosylating enzymes are also expressed at various stages of development and at later stages to regulate multiple processes including transcription, proteolysis, cellular metabolism, and axonal guidance [28]. For example, in adult mice, forebrain specific inactivation of O-GlcNAc transferase (OGT), the enzyme that catalyzes the covalent attachment of N-acetyl-D-glucosamine to serine or threonine residues of proteins, caused neuronal cell death and inflammation [28]. Multiple axon guidance proteins are regulated through glycosylation, directly or indirectly. In the following sections, we discuss N- and O-linked glycosylation of adhesion molecules and other glycoproteins that regulate neurite outgrowth, guidance, and migration.

### 2.1. Glycosylation of Cell Adhesion Molecules in Axonal Guidance

Multiple lines of evidence suggest that perturbation of N-glycosylation leads to defects in cell adhesion and migration. Interactions between glycoproteins and lectins (carbohydrate-binding proteins) are essential for cell-cell communication, signalling, and cell adhesion. Immunoglobulin class cell adhesion molecules (IgCAMs) are glycoproteins that regulate various aspects of cell–cell communication from adhesion, fasciculation, to synapse formation [22]. In this section, we discuss the glycosylation of IgCAMs and their role in axonal guidance.

**Figure 2:**
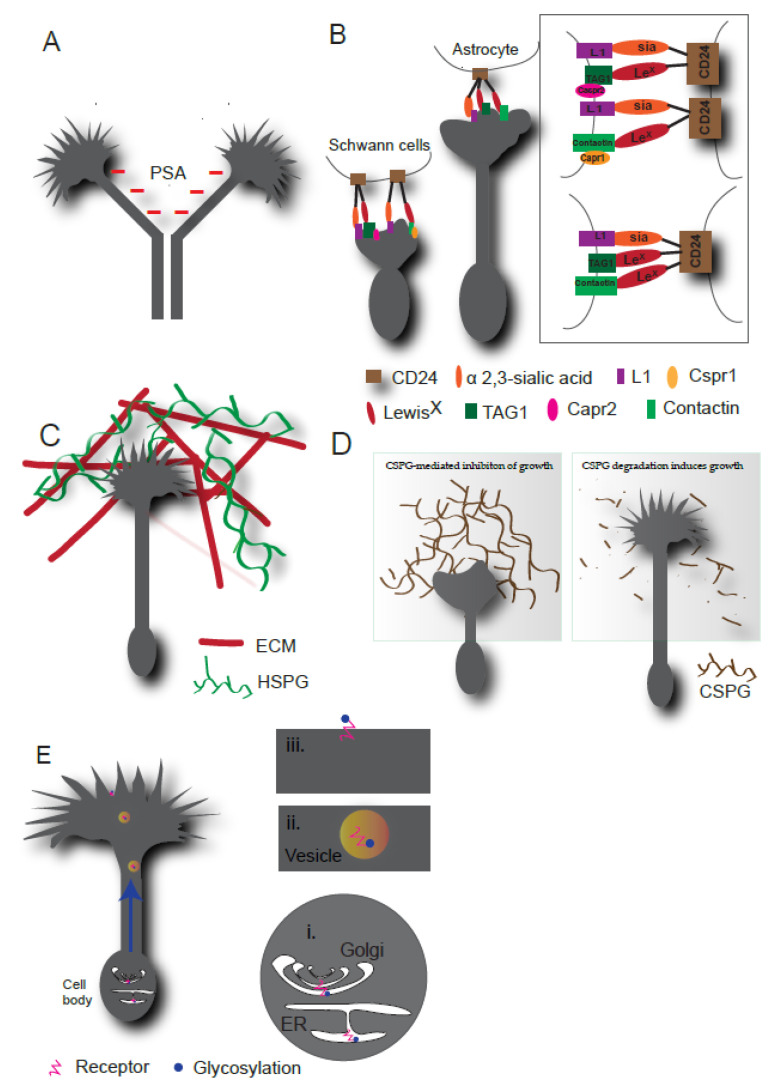
Functions of glycosylation in axon guidance. (**A**) PSA (shown in red) acts as negative regulator of axon fasciculation. (**B**) L1 on neurons binds to α 2,3-sialic acid carried by CD24. Neuronal contactin and TAG1 bind to Lewis^x^ carried by CD24. This CD24, L1, TAG1 and contactin complex causes signal transduction to induce neurite outgrowth in cerebellar neurons (right). In DRG neurons, TAG1 of the TAG1-CD24 complex interacts with Cspr2 and contactin of the contacin-CD24 complex interacts with Cspr1 (left). These two independent complexes cause signal transduction to inhibit neurite outgrowth (box in the right corner shows all the complexes, top: outgrowth inhibiting complex, bottom: outgrowth promoting complex. (**C**) HSPGs are major components of ECM and can modulate neuronal microenvironment by organizing ECM and guidance molecules. (**D**) CSPGs form an inhibitory zone around neurons (left), enzymatic removal CSPGs causes the reversal of inhibition and promotes the growth. (**E**) Role of glycosylation in receptor trafficking and surface representation. On right, (i) ER-Golgi transition (ii) vesicle containing glycosylated receptor and (iii) surface representation of glycosylated receptor are shown. Multiple proteins bind and form a complex with PSA to regulate their functions. Cell surface-exposed PSA interacts with morphogenic factors such as BDNF, NGF, and NT3, and activate their receptors. PSA-bound BDNF leads to activation of BDNF receptors TrkB and p75NTR, resulting in increased survival and growth of neuroblastoma cells [37]. Apart from the NCAM-mediated adhesion by PSA discussed above, PSA has multiple binding partners in the nervous system that regulate processes from neurogenesis to synapse formation in the developing nervous system [24]. However, detailed molecular mechanisms of how PSA modulates these functions need to be investigated to understand higher-order functions of PSA in the nervous system.

Neuronal cell adhesion molecule (NCAM) is a widely expressed IgCAM in the nervous system. NCAM functions in olfactory lobe development, as mutations in NCAM led to the formation of undersized olfactory bulbs [29]. NCAM is modified with polysialic acid (PSA), an N-linked polymer of α2-8 linked neuraminic acid [24]. This modification is crucial for NCAM-mediated neuronal functions in olfactory bulb development. The enzymatic removal of PSA from NCAM compromised the migration of olfactory precursors. PSA transfer onto NCAM is carried out by ST8Sia II and ST8Sia IV polysialyltransferases. Consistent with the NCAM mutants, genetic inactivation of these enzymes in mice resulted in defects in olfactory bulb development and neuronal migration, confirming PSA-NCAM functions in axonal guidance and brain connectivity [30,31]. In the chicken visual system, enzymatic removal of PSA led to several projection errors in the optic fiber layer and many axons failed to leave the retina due to the migration defects [32]. In another study, cleavage of PSA from NCAM by endoneuraminidase N resulted in increased fasciculation of chicken spinal ganglia, suggesting that NCAM adhesion is negatively regulated by PSA (Figure 2A) [33]. Loss of PSA is associated with disruption of several additional NCAM-dependent neurodevelopmental processes outside of the visual and olfactory system. For example, PSA-NCAM functions in hippocampal mossy fiber projections and cortical neuron migration [34]. In the mouse hippocampus, mossy fibers were misrouted in the pyramidal cell layer upon removal of PSA or genetic deletion of PSA-NCAM. The aberrant innervation of mossy fibers was proposed to be due to the excessive adhesion between mossy fibers and the pyramidal cell layer [34]. Loss of PSA resulted in defects in radial and tangential migration of neuronal precursor cells during cortical development in mouse brain; many of these phenotypes were rescued in NCAM deficient mice indicating PSA-NCAM functions in vivo [35]. PSA was predicted to negatively regulate adhesion due to heavy negative charges on its structure. Confirming this, a structural study demonstrated that PSA negatively regulates adhesion by providing steric repulsion. In this study, electron density profiles of NCAM and PSA-NCAM on lipid membranes showed that PSA-NCAM was significantly thicker than NCAM, indicating that PSA increased spacing between adjacent cells and negatively regulates their adhesion [36]. Taken together, PSA regulates NCAM-mediated fasciculation, neuronal migration, and guidance, and therefore is critical for neuronal development.

Several cell surface molecules not only carry glycan structures but also act as acceptors or donors of carbohydrates and regulate surface glycan diversity. L1CAM or L1 is one such example of an IgCAM family glycoprotein that regulates surface glycosylation and downstream cellular functions [38]. In L1 deficient mouse cerebellar granule neurons, mRNA levels of the major glycosylation enzymes, sialyltransferase ST6Gal1, and fucosyltransferase FUT9, were downregulated and cell surface sialylation and fucosylation were reduced. In this study, an L1-specific antibody was used to mimic the L1-L1 trans interaction. This antibody bound to L1 receptors and led to the enhanced outgrowth of L1-expressing neurites. Further, downregulation of ST6Gal1 and FUT9 blocked L1 antibody-mediated outgrowth and migration. Collectively, this study demonstrated that L1 regulates outgrowth and cell migration via regulation of cell surface glycosylation [38]. L1 also regulates neurite outgrowth through heterophilic interactions in trans with CD24, a highly glycosylated mucin glycoprotein. Sialylated CD24 interacted with L1-CAM in trans and promoted neurite outgrowth of mouse cerebellar neurons and inhibited neurite outgrowth of dorsal root ganglion neurons (Figure 2B) [39]. Later, the glycosylation pattern of CD24 was determined for glia-specific isoforms. CD24 exhibited α 2-3 linked sialic acid and Lewis^x^ on its structure. α 2-3 linked sialic acid-L1 trans interaction, Lewis^x^ trans interaction with TAG1 and contactin formed a complex that led to the neurite outgrowth of cerebellar neurons. In DRG neurons, additional cis interactions on neuronal surfaces between contactin and Caspr1 and TAG1 and Casper2 (Figure 2B) led to signal transduction that inhibited the outgrowth. Interestingly, glycosylation on contactin was predicted to regulate its binding to Caspr1. Thus, glycosylation might engage different protein complexes in a cell-specific manner to regulate downstream cellular responses [40].

Multiple other IgCAMs are regulated via glycosylation including SynCAM (Synaptic cell adhesion molecule), DSCAMs (Down Syndrome Cell Adhesion Molecule) and, NrCAM (neural-glia related cell adhesion molecule). SynCAM was initially identified for its involvement in synapse formation [41]. SynCAM also functions as a guidance cue in the central and peripheral nervous system [41]. Similar to NCAM, SynCAM is polysialylated and polysialylation of synCAM has a role in integrating NG2 cells, a glial subpopulation that carries PSA-SynCAM, in murine neuronal networks [42]. However, PSA-mediated regulation of SynCAM in axonal guidance has not been reported. DCSAM is another IgCAM expressed in the developing brain and its expression correlates with neuronal differentiation [43]. Crystal structure analysis suggested that DSCAM1 undergoes homodimerization and N-glycosylation of DSCAM1 regulates dimer formation. How glycosylation eventually affects homophilic recognition of DSCAM and downstream functions remains to be determined [44].

As discussed so far, multiple cell adhesion proteins are glycosylated, but very little is known on how defects in glycosylation lead to CDG. A recent proteomic study showed that cerebellar-specific knockout of *Srd5a3* in mice, a gene involved in the initiation of N-glycosylation, causes hypoglycosylation of multiple glycoproteins. Among these proteins, members of IgCAM family, L1 and NrCAM were hypoglycosylated upon *Srda3* deletion. This reduced the surface localization of L1 and NrCAM and further caused defects in neurite outgrowth and axonal guidance. This study proposed that Srda3-associated and other CDGs are related to hypoglycosylation of multiple proteins, including adhesion proteins [45]. In the future, understanding how multiple glycosylation events affect neuronal migration, neurite outgrowth, and axonal guidance may provide insights for developing CDG models and therapeutic strategies.

Aforementioned studies highlight glycosylation of adhesion molecules and cell surface receptors that regulate neuronal migration, neurite outgrowth, and axon guidance. Glycosylation of signalling components could also potentially regulate such pathways. In the olfactory epithelium, odorant receptor stimulation leads to the generation of cyclic AMP signals and regulation of guidance molecule expression. cAMP is a common secondary messenger in axonal guidance synthesized by adenylyl cyclase 3 (AC3). In olfactory sensory neurons, the activity of cAMP is regulated by glycosylation; cAMP is decorated with poly-*N*-acetyllactosamine (PLN) oligosaccharides. Inactivation of the enzyme that synthesizes PLN led to defects in olfactory bulb innervations, similar to AC3 inactivation. AC3 localization and function was also dependent on N-glycosylation, further highlighting glycosylation function in regulating secondary messenger signalling and axonal guidance [46].

### 2.2. Glycosylation of Dystroglycan in Axonal Guidance

Dystroglycan is a transmembrane protein with a heavily glycosylated extracellular subunit [47,48]. Intracellularly, dystroglycan interacts with actin-binding proteins, suggesting that dystroglycan links the ECM and actin machinery to regulate neuronal migration and growth cone motility [47]. Dystroglycan interacts with multiple guidance proteins and is required for their precise patterning [49,50]. Precise localization of guidance cues is necessary for accurate axonal guidance and downstream functions. Mouse mutants of dystroglycan and its glycosylating enzymes phenocopied spinal commissural guidance defects of slit and robo mutants. Further, binding of glycosylated dystroglycan to slit was shown to be required for the localization of slit within the basement membrane of the floor plate. Glycosylated dystroglycan also regulates axonal guidance by the organization of ECM components in basement membranes [49]. Glycosylated dystroglycan was proposed to serve as a scaffold for multiple proteins including laminin, perlcan, and collagen IV in the basement membrane [47]. Dystroglycan helps maintain the basement membrane in the optic chiasm and was required for axonal sorting of contralateral and ipsilateral optical tracts. A more recent study identified Celsr3, a transmembrane neuronal receptor for dystroglycan, and showed that the Celsr3 interaction with dystroglycan was critical for axonal guidance in vivo. This novel interaction was crucial for the proper rostral movement of commissural axons [47]. The aberrant glycosylation of dystroglycan leads to muscular dystrophy collectively known as dystroglycanpathies [51]. Severe dystroglycanopathies are often associated with neurodevelopmental abnormalities. Whether guidance defects seen in dystroglycan mutants contribute to dystroglycanopathies is an excellent question and needs further studies.

### 2.3. Glycosylation of Endoglycan in Axonal Guidance

Endoglycan is a single-pass transmembrane protein of the CD34 family of sialomucins (mucin with sialic acid on its domain) with extracellular domains harboring many glycosylation sites. A recent study has suggested that endoglycan regulates spinal commissural guidance through its glycosylation. The extracellular domains of sialomucins are highly negatively charged due to N and O-linked glycosylation and multiple sialylations. Downregulation of endoglycan in chicken spinal cord led to increased adhesion between floor plate and commissural axons, axons turned within the floor plate and failed to exit floor plate and move rostrally. This study indicated that endoglycan negatively regulates adhesion at midline similar to what was described for NCAM-PSA in other brain regions [33]. To confirm this, stable expression of endoglycan by monolayers of HEK293 cells were used to study endoglycan mediated adhesion. The enzymatic removal sialic acid and O-glycan from HEK293 cells led to reduced adhesion compared to the control counterparts confirming anti-adhesive functions of endoglycan. [52]. Since endoglycan regulates spinal commissural axons post-crossing (Figure 1B), it will be interesting to see if slit-mediated repulsion of post-crossing commissural axons is regulated by endoglycan. 

## 3. Glycosaminoglycans (GAGs) in Axonal Guidance

GAGs comprise unbranched polysaccharides of repeating disaccharide units that are attached to proteins to form proteoglycans. Long sugar chains are generally repeats of disaccharides formed by GlcNAc or GalNAc, combined with uronic acid or galactose. GAGs can be classified into several subgroups: heparan sulfate (HS), chondroitin sulfate (CS), keratin sulfate (KS), and hyaluronan (HA) [17]. CS, HS, and KS have sulfate groups at different positions and HA is a non-sulfated glycosaminoglycan. Covalent attachment of CS, HS, and KS to proteins form CS proteoglycan (CSPGs), HS proteoglycan (HSPGs), and KS proteoglycan (KSPGs), respectively. HA is considered to be not attached to any core protein [53]. The synthesis of GAG have been reviewed extensively [23] and is not discussed here.

The ECM in the CNS is rich in GAGs and GAGs regulate spatial patterning of many guidance cues in ECM. Though GAGs were initially considered to form a passive physical barrier for inhibiting the growth of a certain population of neurons, several studies have established that GAGs play a role in the diversification of axonal guidance and neuronal plasticity [53]. Interestingly, deletion of enzymes involved in the formation of GAGs are associated with defects in nerve formation and connectivity [23]. Here, we systematically discuss the current understanding of the role of each GAG in regulating axonal guidance pathways.

### 3.1. Hyaluronan (HA) in Axonal Guidance

In the central nervous system, HA is present in interstitial spaces surrounding neurons and astrocytes [54]. HA is a crucial component of brain ECM and perturbation of HA synthase, the enzyme required for HA production, is associated with cognitive impairment and dementia [55]. HA is a major CD44 ligand and shown to be colocalized with CD44 at the optic chiasm. Perturbation of the CD44-HA interaction by administration of exogenous HA caused multiple guidance errors at the midline in mouse optic chiasm [56]. Perturbation of endogenous HA phenocopied these midline crossing defects, suggesting there is regulation of axonal guidance by HA, possibly via its interaction with CD44 [54,56]. HA function in neuronal projections has also been proposed in other neuronal tissues. In the developing brain, afferent fibers terminate at the specific layers in the hippocampus; innervation of entorhinal fibers to the hippocampus is one such example. Treatment of rat hippocampal cultures with hyaluronidase caused defects in fiber projection in the hippocampus, suggesting that HA functions in nerve formation and guidance in this brain region [57]. HA occupies significant real estate in the extracellular space and has been associated with cognitive functions [54]. However, whether it modulates axon guidance molecules and their regulation needs to be investigated further.

### 3.2. Heparan Sulfate Proteoglycan (HSPGs) in Axonal Guidance

HSPGs are major components of the ECM (Figure 2C) and are essential to diversifying cellular microenvironments. Studies in *C. elegans* suggested that lack of HSPG modifying enzymes lead to severe axon guidance defects [58]. Similarly, a wide variety of HSPGs function in the developing *Drosophila* CNS, in processes like fasciculation and axonal guidance, including midline crossing of commissural axons [59,60]. There are multiple HSPG enzymes studied so far that regulate axonal guidance functions [61,62]. HSPGs regulate classic guidance cues including netrins, slit, semaphorins, and ephrins. We discuss HSPG mediated regulation of classic guidance cues in the following section.

#### 3.2.1. Regulation of Slit by HSPGs

The repulsive cue, slit is secreted at the ventral midline, where it prevents the growth of a population of axons expressing its cognate receptor roundabout (Robo) [10]. Syndecan is the major family of transmembrane HSPGs that regulates ECM localization of slit. This is evident by the lack of slit localization on axonal fascicles in syndecan mutants in *Drosophila* [63]. Interestingly, *Mmy* was predicted to carry out glycosylation of syndecan. *Mmy* encodes UDP-N-acetylglucosamine pyrophosphorylase, the enzyme involved in N-linked glycosylation of many proteins [64]. This study showed that glycosylation of slit was essential for its secretion at the ventral midline. Robo is expressed on the longitudinal tracts of axons that run parallel to midline and slit at the ventral midline prevents their crossing. In *Mmy* mutants, distribution and abundance of robo on longitudinal tract were compromised. Reduced levels of slit in *Mmy* mutants may have caused the sparse distribution of Robo. However, the abundance of robo was not reduced in a slit null mutant, suggesting that mmy maintains robo independently of slit secretion [64]. Whether *Mmy* mediated glycosylation regulates sydecan-mediated slit-robo signalling directly remains to be investigated.

Another HSPG that regulates slit-mediated repulsion is glypican. Glypicans are membrane-linked proteins with multiple HS chains attached to their extracellular region [65]. Glypican is one of the major HSPGs of the nervous tissue and regulates axonal guidance. Slits were originally purified as a protein that bind to glypican-1 [66]. Treatment with heparinase III, an enzyme that abolishes HS, disrupted the slit-Robo interaction and chemorepulsion [67]. Additional evidence suggesting HSPG regulated slit-mediated repulsion came from disruption of the gene *Ext1*, involved in polymerization of sugar chains in HS. *Ext1* disruption led to the absence of an olfactory bulb, major defects in commissural tracts, and disruption of cerebellar architecture [60]. Ext2 and Ext3 are other glycosyltransferases that are implicated in HS synthesis. Double mutation genes encoding ext2 and ext3 caused defects in retinotectal projections and phenocopied *Robo2* mutants [23]. These multiple lines of evidence through perturbation of different HSPGs point towards the functions of HSPG in regulating slit mediated axon guidance. Differences in HS-chains (as discussed below) of these HSPGs might be crucial for specific interaction with slit and slit mediated axonal guidance.

Multiple studies highlight more subtle functions of HS synthesis in slit-mediated axonal guidance. The sulfation of HSPG is one such example. Deletion of two *sulfate sulfotransferases* (*Hst*) enzymes, hs2st and hs6st that add a sulfate group to the 2-O position of uronic acid and 6-O position of glucosamine, respectively, resulted in guidance errors in the optic chiasm and corpus callosum. Unlike phenotypes of *Ext* knockouts, wherein HS synthesis was compromised throughout the brain causing altered brain morphology (e.g., lack of olfactory bulb and cerebellar architecture), sulphation mutants showed normal brain morphology. This study highlighted how distinct sulfation patterns on HSPG modulate the axonal guidance. Further in vitro studies suggested that retinal ganglion cell (RGC) axons of *Hs6st1* null mice were less sensitive to Slit2 repulsion than their wild-type counterparts [68]. Collectively HSPG synthesis and sulfation play a crucial role in slit-mediated axonal guidance and functions.

#### 3.2.2. Regulation of Netrin-1 by HSPGs

Netrin-1 is a classic guidance molecule that induces both chemoattraction and chemorepulsion in several different projections [6]. Though classically considered a chemotactic cue, recently haptotactic functions of netrins have been proposed [6,9]. *Ntn1* deletion in vivo is associated with several axon projection defects including agenesis of the corpus callosum, misprojection of spinal and hippocampal commissures, and embryonic lethality in mice. These phenotypes highlight the critical functions of netrin-1 in neurodevelopment [69]. Netrins are required for various functions in addition to axon guidance, including axon branching [6], synaptogenesis [70], and axonal regeneration [71].

The first netrin-1 purification was accomplished by heparan affinity chromatography [72], indicating netrin interacts with heparan. X-ray crystallography of the netrin-1-DCC complex revealed netrin binding to sulfate on heparan [73]. Selective perturbation of Ext-1, the enzyme involved in polymerization of sugar chains in HS, led to commissural midline crossing defects that phenocopied the *Dcc* and *Ntn1* knockouts [74]. Recently, the HSPG glypican was shown to modulate UNC6/netrin mediated axonal guidance by interacting with the netrin receptor UNC40/DCC on the cell surface in *C. elegans* [75]. Since netrin and its receptors are evolutionarily conserved, mammalian netrin-1 may potentially be regulated by glypican and HS synthesis. Glypican might have a potential role in generating diverse growth cone responses; however, detailed mechanisms of glypican-mediated netrin responses remain to be investigated.

#### 3.2.3. Regulation of Semaphorins by HSPGs

Semaphorins are one of the classic guidance cues that function in neuronal migration, proliferation, axonal guidance, and synapse formation. Semaphorins are grouped into seven classes, Class 1 and 2 and Sema5c are found in invertebrates and class 3–7 are present in vertebrates [76]. Semaphorins were originally considered repulsive cues, but later studies also suggested attractive functions of semaphorins. Semaphorin 3A (sema 3A) is a secreted protein that binds to co-receptors neuropilin and plexin during axonal navigation. Secreted sema3A was proposed to bind components of ECM, including GAGs. In vitro cultures of GFP-sema3A-expressing neuro2-A cells demonstrated that sema3A bound to cell surfaces and glass in the surrounding environment. Further, it was shown that sema3A binding was reduced upon HSPG treatment. Interestingly, CSPG also showed similar binding properties to sema3A. HS was shown to increase sema3A binding with neuropilin, causing growth cone collapse [77]. In vivo functions of semaphorin are regulated by multiple proteoglycans. For example, sema5A (discussed in CSPG section) interacts with both HSPG and CSPG.

#### 3.2.4. Regulation of Ephrins by HSPG

Ephrins are receptor tyrosine kinases essential in neurodevelopmental processes including axon guidance [78]. Ephrins are classified into two families: Ephrin A members are linked to the plasma membrane via GPI linkages, whereas ephrin B family members are transmembrane proteins [79]. Disruption of HS by *Ext1* deletion also affected ephrin functions, similar to other guidance cues discussed above. In *Ext1* deleted neurons, ephrin A-mediated growth cone collapse was abolished, highlighting the HS-mediated regulation of ephrin A signalling [80]. However, the molecular details of HS-mediated regulation of ephrin A are unknown. Recent elegant work suggested that a specific N-glycosylation of ephrin B attenuated response of corpus callosum axons to semaphorins. Pre-crossing, semaphorins attracted callosal axons toward the midline. Post-crossing, upregulation of ephrin B attenuated response to semaphorins and the specific glycosylation of ephrin B played a regulatory role in this switch [81]. This demonstrates that HSPG can modulate crosstalk between guidance cues to fine-tune axonal guidance.

### 3.3. Chondroitin Sulfate Proteoglycan (CSPGs) in Axonal Guidance

CSPGs are enriched in the nervous system ECM and function as repulsive factors. The inhibitory role of CSPG in pathfinding was established by several independent studies through its enzymatic removal using chondroitinase ABC. For example, enzymatic removal of CS in mouse brains caused several axon navigation errors [82,83]. The removal of CSPGs from different stages of embryonic development caused a deviation in retinal projections at the midline of the optic chiasm. RGCs project in a region that normally does not support their projection, highlighting inhibitory functions of CSPGs for accurate projections [82]. Similarly, in the zebrafish embryo, injection of chondroitinase ABC before motor axon outgrowth caused abnormal outgrowth of the ventral motor nerve, indicating inhibitory activity of CSPGs [83]. Similar inhibitory functions of CSPGs have also been documented in RGC projections of *Xenopus* embryos and chick embryos [84,85]. A recent in vitro study showed the direct inhibitory effects of CSPGs on neurite outgrowth and growth cone area (Figure 2D) of mouse cerebellar granule neurons [86]. Interestingly, the inhibition of CSPGs was still intact in the presence of growth-promoting laminin. This was in contradiction with the earlier study that showed integrin activation through laminin could revert inhibitory effects of CSPG [87]. How CSPG modulates different guidance molecules, ECM components, signalling pathways, and axon pathfinding is far from a complete understanding. A piece of indirect evidence for the regulation of sema3A by CSPG indicated that CSPG organizes sema-3A in ECM. Brain ECM can be organized in a diffused form or a condensed form, comprising a lattice-like structure called perineuronal nets (PNN). PNN forms around certain subpopulations of neurons and regulate them. Both CSPGs and HSPGs are essential components of PNN. Interestingly, chemorepulsive sema3A decorates the PNN and enzymatic removal of CSPGs disrupted sema3A positive PNN structures. This study suggested that PNN formed by GAGs might pattern guidance cues in PNN and hence regulate axonal guidance [88]. However, functional aspects of PNN formation and its CSPG association needs further investigation.

Another example of the guidance molecule being regulated by CSPGs is Semaphorin 5A (sema5A). Sema5A is a transmembrane protein that can act as both an attractive and repulsive cue [23]. Sema5A physically interacts with both HSPGs and CSPGs and this interaction was required for its guidance function. Rat habenula neurons growing on the stripes of sema5A membrane preparations of HEK293 cells showed preferential growth toward sema5A. Further, precoating of CSPG of stripes preparations converted sema5A from an attractive to repulsive cue. Interestingly, CSPGs have the unique function of converting sema5A from attractive to inhibitory cue and HSPG was shown to be required for sema5A-mediated attraction [89]. This shows how different GAGs generate the diversity of responses to fine-tune axonal pathfinding.

CSPGs have an inhibitory effect on the growth and regeneration of neurons in the adult nervous system. Often nerve injury leads to ECM damage and growth inhibitory CSPG upregulation upon injury [90]. CSPG is an excellent candidate to manipulate regeneration. Expression of the specific CSPG interactors that reverse the CSPG mediated inhibition might promote axon regeneration. One such example of a CSPG binding partner is heparan-binding growth-associated molecule (HB-GAM) that was shown to induce neuronal outgrowth in the presence of inhibitory CSPGs. Post-injury injection of HB-GAM in mice spinal cord caused a significant increase in axon regeneration compared to control, confirming in vitro findings [91]. Screening of such growth factors and antagonists of CSPGs might be important in regeneration therapies.

## 4. Conclusions and Future Challenges

**Figure 3:**
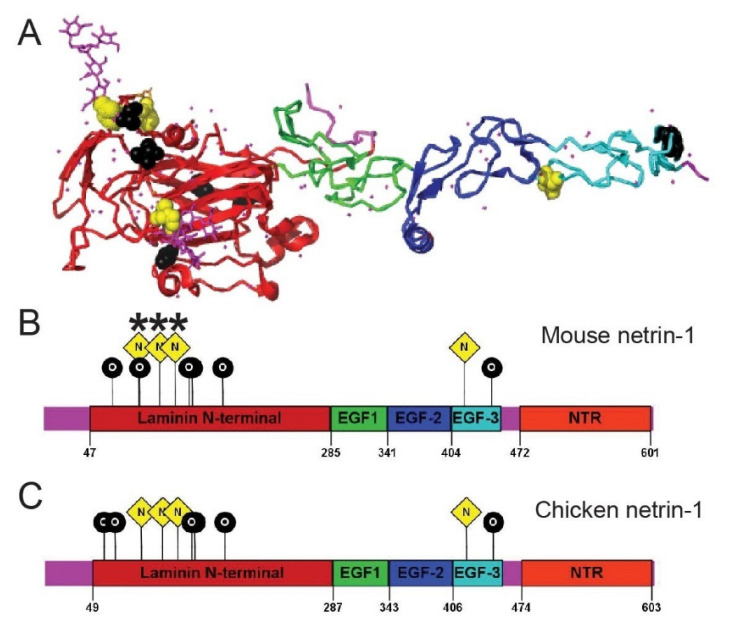
Glycosylation sites prediction of netrin-1 (**A**) Crystal structure of mouse netrin-1 generated using PyMOL using PDB files obtained from UniProt. Glycosylation sites predicted from GlycoMine ^struct^ [92] are presented with black and yellow spheres in crystal structure. Yellow and black spheresindicate N-linked (probability more than 0.8) and l O-linked (probability more than 0.95) glycosylation sitesrespectively. (**B**,**C**) Domain organization of mouse (**B**) and chicken (**C**) netrin-1 with predicted glycosylations sites. N-linked glycosylation sites known from the crystal structure are marked with asterisks (N91, N116, and N131). Color code of domains is consistent with structure shown in A (NTR domain is not represented in A).

The formation of glycan conjugates with proteins is a multistep process that regulates protein functions including protein folding, stability, trafficking, secretion (Figure 2E), and specific interactions. Glycoconjugation is an essential part of generating functional diversity during neurodevelopment. Multiple studies show that the inactivation of glycosidases and glycosyl transferases culminate in defects in axonal guidance and brain development. Further, brain tissue wide understanding of expression patterns of these enzymes, their substrates, and the impact of glycosylation in cell migration, pathfinding, and synapse formation have contributed significantly towards the current understanding of the field [21]. However, the removal of enzymes is likely to affect multiple substrates, complicating the interpretation of such studies. Hence, in the future, candidate-based approaches along with high throughput studies are needed to clarify the function of specific glycosylation events. This remains challenging since glycosylation is a non-templated modification. Bioinformatic tool like GlycoMine^struct^ that predict N and O glycosylation based on the sequence and structural data would be useful for primary screening [92]. For example, structural data from netrin-1 UNC5H2 complex directly shows three N-linked glycosylation sites (Asn95, Asn116, and Asn131) [92,93]. However, bioinformatic analysis using GlycoMine^struct^ predicted multiple additional glycosylation sites (Figure 3). Additionally, there could be potential glycosylation sites in the unstructured region of proteins that may not be captured in the structural analysis. Thus, multiple complementary approaches are needed to fully predict glycosylation pattern and its functions. This includes mass spectroscopic analysis of glycoconjugates, lectin binding, or glycan-binding chips for high throughput screening. Further, subtle and specific perturbations will allow dissection of molecular mechanisms and functional consequences of glycosylation [94,95]. Analysis of the glycosylated proteome of brain tissue has revealed myriad glycosylated candidates with roles in neurodevelopment [19]. There are multiple pieces of evidence suggesting altered glycosylation occurs after injury, and hence process of glycosylation can be targeted for axon regeneration [96]. The development of novel biomarkers for glycosylation might serve as essential diagnostic tools in many CDGs. In depth understanding of glycosylation within the brain and spinal cord might elevate the number of candidates for regeneration therapies.

## References

[1] Comer J., Alvarez S., Butler S., Kaltschmidt J. (2019). Commissural axon guidance in the developing spinal cord: From Cajal to the present day. Neural Dev..

[2] Stoeckli E. (2017). Where does axon guidance lead us?. F1000Research.

[3] Lowery L.A., Van Vactor D. (2009). The trip of the tip: Understanding the growth cone machinery. Nat. Rev. Mol. cell Biol..

[4] Bellon A., Mann F. (2018). Keeping up with advances in axon guidance. Curr. Opin. Neurobiol..

[5] McCormick L.E., Gupton S.L. (2020). Mechanistic advances in axon pathfinding. Curr. Opin. Cell Biol..

[6] Boyer N.P., Gupton S.L. (2018). Revisiting Netrin-1: One who guides (axons). Front. Cell. Neurosci..

[7] Stoeckli E.T. (2018). Understanding axon guidance: Are we nearly there yet?. Development.

[8] Wu Z., Makihara S., Yam P.T., Teo S., Renier N., Balekoglu N., Moreno-Bravo J.A., Olsen O., Chédotal A., Charron F. (2019). Long-range guidance of spinal commissural axons by netrin1 and sonic hedgehog from midline floor plate cells. Neuron.

[9] Varadarajan S.G., Kong J.H., Phan K.D., Kao T.-J., Panaitof S.C., Cardin J., Eltzschig H., Kania A., Novitch B.G., Butler S.J. (2017). Netrin1 produced by neural progenitors, not floor plate cells, is required for axon guidance in the spinal cord. Neuron.

[10] Araújo S.J., Tear G. (2003). Axon guidance mechanisms and molecules: Lessons from invertebrates. Nat. Rev. Neurosci..

[11] Vysokov N.V., Silva J.-P., Lelianova V.G., Suckling J., Cassidy J., Blackburn J.K., Yankova N., Djamgoz M.B., Kozlov S.V., Tonevitsky A.G. (2018). Proteolytically released Lasso/teneurin-2 induces axonal attraction by interacting with latrophilin-1 on axonal growth cones. Elife.

[12] Guy A.T., Nagatsuka Y., Ooashi N., Inoue M., Nakata A., Greimel P., Inoue A., Nabetani T., Murayama A., Ohta K. (2015). Glycerophospholipid regulation of modality-specific sensory axon guidance in the spinal cord. Science.

[13] Ye X., Qiu Y., Gao Y., Wan D., Zhu H. (2019). A subtle network mediating axon guidance: Intrinsic dynamic structure of growth cone, attractive and repulsive molecular cues, and the intermediate role of signaling pathways. Neural Plast..

[14] Chédotal A. (2019). Roles of axon guidance molecules in neuronal wiring in the developing spinal cord. Nat. Rev. Neurosci..

[15] Seiradake E., Jones E.Y., Klein R. (2016). Structural perspectives on axon guidance. Annu. Rev. cell Dev. Biol..

[16] He W., Wei L., Zou Q. (2019). Research progress in protein posttranslational modification site prediction. Brief. Funct. Genom..

[17] Higuero A.M., Díez-Revuelta N., Abad-Rodríguez J. (2017). The sugar code in neuronal physiology. Histochem. Cell Biol..

[18] Reily C., Stewart T.J., Renfrow M.B., Novak J. (2019). Glycosylation in health and disease. Nat. Rev. Nephrol..

[19] Fang P., Wang X.-J., Xue Y., Liu M.-Q., Zeng W.-F., Zhang Y., Zhang L., Gao X., Yan G.-Q., Yao J. (2016). In-Depth mapping of the mouse brain N-Glycoproteome reveals widespread N-Glycosylation of diverse brain proteins. Oncotarget.

[20] Ohtsubo K., Marth J.D. (2006). Glycosylation in cellular mechanisms of health and disease. Cell.

[21] Stanley P. (2016). What have we learned from glycosyltransferase knockouts in mice?. J. Mol. Biol..

[22] Scott H., Panin V.M. (2014). N-glycosylation in regulation of the nervous system. Glycobiology of the Nervous System.

[23] Masu M. (2016). Proteoglycans and axon guidance: A new relationship between old partners. J. Neurochem..

[24] Sytnyk V., Leshchyns’ka I., Schachner M. (2020). Neural glycomics: The sweet side of nervous system functions. Cell. Mol. Life Sci..

[25] Dong X., Huang Y., Cho B.G., Zhong J., Gautam S., Peng W., Williamson S.D., Banazadeh A., Torres-Ulloa K.Y., Mechref Y. (2018). Advances in mass spectrometry-based glycomics. Electrophoresis.

[26] Ye Z., Marth J.D. (2004). N-glycan branching requirement in neuronal and postnatal viability. Glycobiology.

[27] Lee J., Ha S., Kim M., Kim S.-W., Yun J., Ozcan S., Hwang H., Ji I.J., Yin D., Webster M.J. (2020). Spatial and temporal diversity of glycome expression in mammalian brain. Proc. Natl. Acad. Sci. USA.

[28] Wang A.C., Jensen E.H., Rexach J.E., Vinters H.V., Hsieh-Wilson L.C. (2016). Loss of O-GlcNAc glycosylation in forebrain excitatory neurons induces neurodegeneration. Proc. Natl. Acad. Sci. USA.

[29] Ono K., Tomasiewicz H., Magnuson T., Rutishauser U. (1994). N-CAM mutation inhibits tangential neuronal migration and is phenocopied by enzymatic removal of polysialic acid. Neuron.

[30] Weinhold B., Seidenfaden R., Röckle I., Mühlenhoff M., Schertzinger F., Conzelmann S., Marth J.D., Gerardy-Schahn R., Hildebrandt H. (2005). Genetic ablation of polysialic acid causes severe neurodevelopmental defects rescued by deletion of the neural cell adhesion molecule. J. Biol. Chem..

[31] Hildebrandt H., Mühlenhoff M., Oltmann-Norden I., Röckle I., Burkhardt H., Weinhold B., Gerardy-Schahn R. (2009). Imbalance of neural cell adhesion molecule and polysialyltransferase alleles causes defective brain connectivity. Brain.

[32] Monnier P.P., Beck S.G., Bolz J., Henke-Fahle S. (2001). The polysialic acid moiety of the neural cell adhesion molecule is involved in intraretinal guidance of retinal ganglion cell axons. Dev. Biol..

[33] Rutishauser U., Watanabe M., Silver J., Troy F.A., Vimr E.R. (1985). Specific alteration of NCAM-mediated cell adhesion by an endoneuraminidase. J. Cell Biol..

[34] Koyama R., Ikegaya Y. (2018). The molecular and cellular mechanisms of axon guidance in mossy fiber sprouting. Front. Neurol..

[35] Angata K., Huckaby V., Ranscht B., Terskikh A., Marth J.D., Fukuda M. (2007). Polysialic acid-directed migration and differentiation of neural precursors are essential for mouse brain development. Mol. Cell. Biol..

[36] Rutishauser U., Landmesser L. (1996). Polysialic acid in the vertebrate nervous system: A promoter of plasticity in cell-cell interactions. Trends Neurosci..

[37] Kanato Y., Kitajima K., Sato C. (2008). Direct binding of polysialic acid to a brain-derived neurotrophic factor depends on the degree of polymerization. Glycobiology.

[38] Li Y.-L., Wu G.-Z., Dawe G.S., Zeng L., Cui S.-S., Loers G., Tilling T., Sun L., Schachner M., Xiao Z.-C. (2008). Cell surface sialylation and fucosylation are regulated by L1 via phospholipase Cγ and cooperate to modulate neurite outgrowth, cell survival and migration. PLoS ONE.

[39] Kleene R., Yang H., Kutsche M., Schachner M. (2001). The neural recognition molecule L1 is a sialic acid-binding lectin for CD24, which induces promotion and inhibition of neurite outgrowth. J. Biol. Chem..

[40] Lieberoth A., Splittstoesser F., Katagihallimath N., Jakovcevski I., Loers G., Ranscht B., Karagogeos D., Schachner M., Kleene R. (2009). Lewisx and α2, 3-sialyl glycans and their receptors TAG-1, Contactin, and L1 mediate CD24-dependent neurite outgrowth. J. Neurosci..

[41] Frei J.A., Stoeckli E.T. (2017). SynCAMs–From axon guidance to neurodevelopmental disorders. Mol. Cell. Neurosci..

[42] Galuska S.P., Rollenhagen M., Kaup M., Eggers K., Oltmann-Norden I., Schiff M., Hartmann M., Weinhold B., Hildebrandt H., Geyer R. (2010). Synaptic cell adhesion molecule SynCAM 1 is a target for polysialylation in postnatal mouse brain. Proc. Natl. Acad. Sci. USA.

[43] Agarwala K.L., Ganesh S., Amano K., Suzuki T., Yamakawa K. (2001). DSCAM, a highly conserved gene in mammals, expressed in differentiating mouse brain. Biochem. Biophys. Res. Commun..

[44] Li S.-A., Cheng L., Yu Y., Wang J.-h., Chen Q. (2016). Structural basis of Dscam1 homodimerization: Insights into context constraint for protein recognition. Sci. Adv..

[45] Medina-Cano D., Ucuncu E., Nguyen L.S., Nicouleau M., Lipecka J., Bizot J.-C., Thiel C., Foulquier F., Lefort N., Faivre-Sarrailh C. (2018). High N-glycan multiplicity is critical for neuronal adhesion and sensitizes the developing cerebellum to N-glycosylation defect. Elife.

[46] Henion T.R., Faden A.A., Knott T.K., Schwarting G.A. (2011). β3GnT2 maintains adenylyl cyclase-3 signaling and axon guidance molecule expression in the olfactory epithelium. J. Neurosci..

[47] Lindenmaier L.B., Parmentier N., Guo C., Tissir F., Wright K.M. (2019). Dystroglycan is a scaffold for extracellular axon guidance decisions. Elife.

[48] Moore C.J., Winder S.J. (2010). Dystroglycan versatility in cell adhesion: A tale of multiple motifs. Cell Commun. Signal..

[49] Clements R., Turk R., Campbell K.P., Wright K.M. (2017). Dystroglycan maintains inner limiting membrane integrity to coordinate retinal development. J. Neurosci..

[50] Wright K.M., Lyon K.A., Leung H., Leahy D.J., Ma L., Ginty D.D. (2012). Dystroglycan organizes axon guidance cue localization and axonal pathfinding. Neuron.

[51] Godfrey C., Foley A.R., Clement E., Muntoni F. (2011). Dystroglycanopathies: Coming into focus. Curr. Opin. Genet. Dev..

[52] Baeriswyl T., Dumoulin A., Schaettin M., Tsapara G., Niederkofler V., Helbling D., Avilés E., Frei J.A., Wilson N.H., Gesemann M. (2021). Endoglycan plays a role in axon guidance by modulating cell adhesion. Elife.

[53] Miyata S., Kitagawa H. (2017). Formation and remodeling of the brain extracellular matrix in neural plasticity: Roles of chondroitin sulfate and hyaluronan. Biochim. et Biophys. Acta (BBA)-Gen. Subj..

[54] Lin L., Wang J., Chan C.K., Chan S.O. (2007). Effects of exogenous hyaluronan on midline crossing and axon divergence in the optic chiasm of mouse embryos. Eur. J. Neurosci..

[55] Balashova A., Pershin V., Zaborskaya O., Tkachenko N., Mironov A., Guryev E., Kurbatov L., Gainullin M., Mukhina I. (2019). Enzymatic digestion of hyaluronan-based brain extracellular matrix in vivo can induce seizures in neonatal mice. Front. Neurosci..

[56] Haupt C., Huber A.B. (2008). How axons see their way–axonal guidance in the visual system. Front. Biosci.

[57] Förster E., Zhao S., Frotscher M. (2001). Hyaluronan-associated adhesive cues control fiber segregation in the hippocampus. Development.

[58] Bülow H.E., Hobert O. (2004). Differential sulfations and epimerization define heparan sulfate specificity in nervous system development. Neuron.

[59] Smart A.D., Course M.M., Rawson J., Selleck S., Van Vactor D., Johnson K.G. (2011). Heparan sulfate proteoglycan specificity during axon pathway formation in the Drosophila embryo. Dev. Neurobiol..

[60] Inatani M., Irie F., Plump A.S., Tessier-Lavigne M., Yamaguchi Y. (2003). Mammalian brain morphogenesis and midline axon guidance require heparan sulfate. Science.

[61] Esko J.D., Lindahl U. (2001). Molecular diversity of heparan sulfate. J. Clin. Investig..

[62] Esko J.D., Selleck S.B. (2002). Order out of chaos: Assembly of ligand binding sites in heparan sulfate. Annu. Rev. Biochem..

[63] Johnson K.G., Ghose A., Epstein E., Lincecum J., O’Connor M.B., Van Vactor D. (2004). Axonal heparan sulfate proteoglycans regulate the distribution and efficiency of the repellent slit during midline axon guidance. Curr. Biol..

[64] Manavalan M.A., Jayasinghe V.R., Grewal R., Bhat K.M. (2017). The glycosylation pathway is required for the secretion of Slit and for the maintenance of the Slit receptor Robo on axons. Sci. Signal..

[65] De Pasquale V., Pavone L.M. (2019). Heparan sulfate proteoglycans: The sweet side of development turns sour in mucopolysaccharidoses. Biochim. Biophys. Acta (BBA)-Mol. Basis Dis..

[66] Ypsilanti A.R., Zagar Y., Chédotal A. (2010). Moving away from the midline: New developments for Slit and Robo. Development.

[67] Piper M., Anderson R., Dwivedy A., Weinl C., Van Horck F., Leung K.M., Cogill E., Holt C. (2006). Signaling mechanisms underlying Slit2-induced collapse of Xenopus retinal growth cones. Neuron.

[68] Pratt T., Conway C.D., Tian N.M.-L., Price D.J., Mason J.O. (2006). Heparan sulphation patterns generated by specific heparan sulfotransferase enzymes direct distinct aspects of retinal axon guidance at the optic chiasm. J. Neurosci..

[69] Yung A.R., Nishitani A.M., Goodrich L.V. (2015). Phenotypic analysis of mice completely lacking netrin 1. Development.

[70] Glasgow S.D., Ruthazer E.S., Kennedy T.E. (2021). Guiding synaptic plasticity: Novel roles for netrin-1 in synaptic plasticity and memory formation in the adult brain. J. Physiol..

[71] Dun X.-P., Parkinson D.B. (2017). Role of netrin-1 signaling in nerve regeneration. Int. J. Mol. Sci..

[72] Serafini T., Colamarino S.A., Leonardo E.D., Wang H., Beddington R., Skarnes W.C., Tessier-Lavigne M. (1996). Netrin-1 is required for commissural axon guidance in the developing vertebrate nervous system. Cell.

[73] Finci L.I., Krüger N., Sun X., Zhang J., Chegkazi M., Wu Y., Schenk G., Mertens H.D., Svergun D.I., Zhang Y. (2014). The crystal structure of netrin-1 in complex with DCC reveals the bifunctionality of netrin-1 as a guidance cue. Neuron.

[74] Matsumoto Y., Irie F., Inatani M., Tessier-Lavigne M., Yamaguchi Y. (2007). Netrin-1/DCC signaling in commissural axon guidance requires cell-autonomous expression of heparan sulfate. J. Neurosci..

[75] Blanchette C.R., Perrat P.N., Thackeray A., Bénard C.Y. (2015). Glypican is a modulator of netrin-mediated axon guidance. PLoS Biol..

[76] Alto L.T., Terman J.R. (2017). Semaphorins and their signaling mechanisms. Method Mol. Bio..

[77] De Wit J., De Winter F., Klooster J., Verhaagen J. (2005). Semaphorin 3A displays a punctate distribution on the surface of neuronal cells and interacts with proteoglycans in the extracellular matrix. Mol. Cell. Neurosci..

[78] Klein R. (2004). Eph/ephrin signaling in morphogenesis, neural development and plasticity. Curr. Opin. Cell Biol..

[79] Kolodkin A.L., Tessier-Lavigne M. (2011). Mechanisms and molecules of neuronal wiring: A primer. Cold Spring Harb. Perspect. Biol..

[80] Irie F., Okuno M., Matsumoto K., Pasquale E.B., Yamaguchi Y. (2008). Heparan sulfate regulates ephrin-A3/EphA receptor signaling. Proc. Natl. Acad. Sci. USA.

[81] Mire E., Hocine M., Bazellières E., Jungas T., Davy A., Chauvet S., Mann F. (2018). Developmental upregulation of Ephrin-B1 silences Sema3C/Neuropilin-1 signaling during post-crossing navigation of corpus callosum axons. Curr. Biol..

[82] Chung K., Taylor J., Shum D., Chan S. (2000). Axon routing at the optic chiasm after enzymatic removal of chondroitin sulfate in mouse embryos. Development.

[83] Bernhardt R.R., Schachner M. (2000). Chondroitin sulfates affect the formation of the segmental motor nerves in zebrafish embryos. Dev. Biol..

[84] Walz A., McFarlane S., Brickman Y.G., Nurcombe V., Bartlett P.F., Holt C.E. (1997). Essential role of heparan sulfates in axon navigation and targeting in the developing visual system. Development.

[85] Ichijo H., Kawabata I. (2001). Roles of the telencephalic cells and their chondroitin sulfate proteoglycans in delimiting an anterior border of the retinal pathway. J. Neurosci..

[86] Jin J., Tilve S., Huang Z., Zhou L., Geller H.M., Yu P. (2018). Effect of chondroitin sulfate proteoglycans on neuronal cell adhesion, spreading and neurite growth in culture. Neural Regen. Res..

[87] Tan C.L., Kwok J.C., Patani R., Chandran S., Fawcett J.W. (2011). Integrin activation promotes axon growth on inhibitory chondroitin sulfate proteoglycans by enhancing integrin signaling. J. Neurosci..

[88] Vo T., Carulli D., Ehlert E.M., Kwok J.C., Dick G., Mecollari V., Moloney E.B., Neufeld G., de Winter F., Fawcett J.W. (2013). The chemorepulsive axon guidance protein semaphorin3A is a constituent of perineuronal nets in the adult rodent brain. Mol. Cell. Neurosci..

[89] Kantor D.B., Chivatakarn O., Peer K.L., Oster S.F., Inatani M., Hansen M.J., Flanagan J.G., Yamaguchi Y., Sretavan D.W., Giger R.J. (2004). Semaphorin 5A is a bifunctional axon guidance cue regulated by heparan and chondroitin sulfate proteoglycans. Neuron.

[90] Kim J., Sajid M.S., Trakhtenberg E.F. (2018). The extent of extra-axonal tissue damage determines the levels of CSPG upregulation and the success of experimental axon regeneration in the CNS. Sci. Rep..

[91] Rauvala H., Paveliev M., Kuja-Panula J., Kulesskaya N. (2017). Inhibition and enhancement of neural regeneration by chondroitin sulfate proteoglycans. Neural Regen. Res..

[92] Li F., Li C., Revote J., Zhang Y., Webb G.I., Li J., Song J., Lithgow T. (2016). GlycoMine struct: A new bioinformatics tool for highly accurate mapping of the human N-linked and O-linked glycoproteomes by incorporating structural features. Sci. Rep..

[93] Grandin M., Meier M., Delcros J.G., Nikodemus D., Reuten R., Patel T.R., Goldschneider D., Orriss G., Krahn N., Boussouar A. (2016). Structural decoding of the Netrin-1/UNC5 interaction and its therapeutical implications in cancers. Cancer Cell.

[94] Roy R., Cao Y., Kaltner H., Kottari N., Shiao T.C., Belkhadem K., André S., Manning J.C., Murphy P.V., Gabius H.-J. (2017). Teaming up synthetic chemistry and histochemistry for activity screening in galectin-directed inhibitor design. Histochem. Cell Biol..

[95] Xiao H., Chen W., Smeekens J.M., Wu R. (2018). An enrichment method based on synergistic and reversible covalent interactions for large-scale analysis of glycoproteins. Nat. Commun..

[96] Osimanjiang W., Roballo K.C.S., Houck B.D., Ito M., Antonopoulos A., Dell A., Haslam S.M., Bushman J.S. (2020). Analysis of N-and O-Linked Glycosylation: Differential Glycosylation after Rat Spinal Cord Injury. J. Neurotrauma.

